# Optical near-fields & nearfield optics

**DOI:** 10.3762/bjnano.5.19

**Published:** 2014-02-19

**Authors:** Alfred J Meixner, Paul Leiderer

**Affiliations:** 1 Institute of Physical and Theoretical Chemistry, University of Tübingen, Auf der Morgenstelle 18, 72076 Tübingen, Germany; 2 Physics Department, University of Konstanz, Universitätsstraße 10, 78464 Konstanz, Germany

Optical methods provide exceedingly powerful tools in science and technology for measuring, analyzing and manipulating, from optical microscopy and spectroscopy to the characterization of ultrafast processes by femtosecond pulses and the modification of materials by intense laser radiation. However, when it comes to applications in the nanometer-regime, the conventional optical techniques suffer from the resolution limit – formulated by Ernst Abbe one and a half centuries ago – that light cannot be focused to a diameter much less than half its wavelength. This seems like a serious drawback for the use of optics in nanoscience and nanotechnology, since many of the nanostructures of interest are distinctly smaller than this limit of a few hundred nanometers. Fortunately, this restriction can be overcome, if one does not rely on focusing with lenses and mirrors in the optical far-field, but rather exploits the optical near-field in the vicinity of nanostructures. In this Thematic Series, various examples for the use of optical near-fields and near-field optics are presented.

Metallic nanostructures, especially noble metals such as gold and silver, are efficient for nano-focusing and controlling light on the nanoscale, because they support surface plasmons, i.e., collective excitations of the electron gas, which couple strongly to light. As a result, the optical near-field around such plasmonic structures can be enhanced by orders of magnitude compared to the incident light intensity, and can be localized in “hot spots” with a length of a few nanometers. This effect of strong near-field enhancement around sharp structures of noble metals has been known from Surface Enhanced Raman Scattering (SERS) for a long time [1]. Yet, the well-controlled tailoring of nanostructures necessary to quantitatively control the optical near-field has only emerged a few years ago. In this issue, Katrin and Harald Kneipp [2] address in their contribution the possibility to probe the plasmonic near-fields by one- and two-photon excited surface enhanced Raman scattering at the level of single molecules. In addition to SERS, there are numerous other applications of near-field enhancement, e.g., in biosensors, solar cells and semiconductor quantum dots to name but a few.

A challenging question, investigated in this series by Esmann et al. [3], is how light can be most efficiently coupled into sub-wavelength dimensions by means of an “optical antenna”. Since the fabrication of suitable structures with electron beam or focused ion beam lithography is a tedious and time-consuming task, the experiments are more and more supported by modeling with numerical methods such as Finite Difference Time Domain (FDTD) and Discrete Dipole Approximation (DDA). For the modeling to be reliable, it is essential to quantitatively compare the results of simulations with experimental data. To this end, various schemes have been developed, like Scanning Near-field Optical Microscopy (SNOM) and Photoelectron Electron Microscopy (PEEM), in order to image optical near-fields of nanostructures. Since the field enhancement can be quite large, light-induced local changes of the material can also be utilized to map the spatial distribution of the near-fields as demonstrated by Dickreuter et al. [4]. For this purpose, light-induced local changes of the material of both the nanostructure itself by local melting and the substrate by ablation at the positions of the hot spots may be used.

The interaction of plasmonic structures with their surroundings can be employed to tune their optical properties, e.g., by using a dielectric phase change material like GeSbTe as a substrate, whose refractive index can be externally adjusted in various ways. As shown by Hong et al. [5], this is a promising new approach for nano-circuitry, bio-assay addressing and imaging applications. A particularly intriguing effect, investigated by Arnold et al. [6], is the interaction of plasmonic structures with dielectric material that is doped with fluorescent molecules: when the emission line of the dye and the absorption resonance of the nanostructures coincide, the damping of the plasmons can be compensated by the gain in the dielectric material, so that laser-like radiation could emerge from such structures (so-called spasers).

The mechanical effects of the optical near-fields can be substantial. Examples are specially shaped nano-holes, studied by Rosa et al. [7], which can be much more efficiently used as plasmonic optical tweezers for nano-objects than the usual far-field tweezers.

In spite of the high efficiency of plasmonic metal nanostructures, the near-field enhancement of dielectric structures is preferable for some applications. Walhorn et al. [8] have developed a method for the simultaneous recording of topography and fluorescence that allows for the localization of distinct building blocks of supramolecular structures below the usual optical resolution. This is facilitated by using the optical near-field around a sharp dielectric AFM tip to excite the fluorescent molecules – a metallic tip would quench the fluorescence. The optical near-fields of dielectric objects can also be used to study the interaction of intense laser radiation with surfaces on ultrashort time scales, which avoids complications like self-focusing that arise otherwise, as shown by Kühler et al. [9].

The examples given above illustrate the broad spectrum of applications of optical near-fields and near-field optics. The goal of this Thematic Series is not to provide a comprehensive review of all the aspects of this theme, but rather to highlight several facets in a rapidly developing research field. We sincerely thank all colleagues who contributed to this Thematic Series with their interesting and exciting results.

Alfred J. Meixner and Paul Leiderer

Tübingen, Konstanz, January 2014
